# Malaria and Under-Nutrition: A Community Based Study Among Under-Five Children at Risk of Malaria, South-West Ethiopia

**DOI:** 10.1371/journal.pone.0010775

**Published:** 2010-05-21

**Authors:** Amare Deribew, Fessehaye Alemseged, Fasil Tessema, Lelisa Sena, Zewdie Birhanu, Ahmed Zeynudin, Morankar Sudhakar, Nasir Abdo, Kebede Deribe, Sibhatu Biadgilign

**Affiliations:** 1 Department of Epidemiology, Jimma University, Jimma, Ethiopia; 2 Department of Health Education and Behavioral Sciences, Jimma University, Jimma, Ethiopia; 3 Department of Medical Laboratory and Pathology, Jimma University, Jimma, Ethiopia; 4 Jimma Zone Health Department, Jimma, Ethiopia; 5 Fayyaa Integrated Development Association-NCMI, Addis Ababa, Ethiopia; 6 Johns Hopkins University-Technical Support for the Ethiopian HIV/AIDS ART Initiative, Addis Ababa, Ethiopia; Aga Khan University, Pakistan

## Abstract

**Background:**

The interaction between malaria and under-nutrition is not well elucidated in Ethiopia. The objective of this study was to assess the magnitude of under-nutrition and its correlation with malaria among under-five children in south-west Ethiopia.

**Methods:**

This cross-sectional study was undertaken during March–February, 2009 as part of the baseline assessment of a cluster randomized trial around Gilgel Gibe Hydroelectric dam, south-west Ethiopia. A total of 2410 under-five children were included for anthropometric measurement and blood investigation for the diagnosis of malaria and anemia. The nutritional status of children was determined using the International Reference Population defined by the U.S National Center for Health Statistics (NCHS). Blood film was used to identify malaria parasite and haemoglobin concentration was determined by Hemo Cue analyzer *(HemoCue Hb 301, Sweden)*.

**Results:**

Significant proportion (40.4%) of under-five children were stunted (height-for-age<−2SD). The prevalence of under-weight was 34.2%. One third and one tenth of the children had anemia and malaria parasite respectively. Older children were more likely to have under-nutrition. There was no association between malaria and under-nutrition. Children who had malaria parasite were 1.5 times more likely to become anaemic compare to children who had no malaria parasite, [OR = 1.5, (95% CI: 1.1–2.0)].

**Conclusion:**

In this study, there is no association between malaria and under-nutrition. Children who have malaria are more likely to be anaemic. Malaria prevention and control program should consider nutrition interventions particularly anemia.

## Introduction

Malaria and under-nutrition are the two major causes of childhood mortality in sub-Saharan Africa [1]. Each year, malaria kills more than 800,000 people annually, of which 91% of them reside in Africa and 85% of them are under five children [2]. On the other hand, under-nutrition is considered to be the underlying cause for more than 50% of deaths of under-five children [3]. In Ethiopia, malaria and malnutrition are the top causes of morbidity and mortality in under-five children [2], [4].

The relationship between malaria and under-nutrition is debatable. Although a number of observations have indicated a deleterious effect of malaria on nutritional status [5], [6], it is still unclear whether and how nutritional status influences malaria-related morbidity. Earlier observational studies provide some evidence of protective effect of under-nutrition against malaria [7], [8], [9]. However, more recent studies have presented inconsistent findings. Deen et al in Gambia and Friedman et al in Kenya reported that under-weight was not associated with infection with malaria [10], [11]. Another study in Gambia showed that nutritional status was not associated with the occurrence of malaria [12].

Results on the relationship between malaria and stunting are conflicting. Stunting was the risk factors of malaria in Gambia and Kenya [10], [11]. In contrary, a study in Papua New Guinea showed that stunting protected children from malaria [13].

In Ethiopia, where malaria and malnutrition are the major public health problems, little is known about the interaction between the two diseases. The objective of this study was to assess the effect of malaria parasite on the nutritional status of under-five children who are at risk of malaria around Gilgel Gibe hydroelectric dam, south-west Ethiopia.

## Materials and Methods

The study was conducted in Gilgel Gibe Field Research Center (GGFRC). This research site is selected since malaria is the major health problem in the area due to ecological disruption [14]. GGFRC was established in 2005 to serve as Demographic Surveillance System and field attachment site of Jimma University. The research center comprises of eight rural and two urban *Kebeles* (lowest administration unit in Ethiopia) which are located around the reservoir of Gilgel Gibe hydroelectric dam. In the ten Kebeles, there are 52 *Gots (villages)*, 55,000 population and 10800 households.

This cross-sectional study was undertaken as part of the baseline assessment of a cluster randomized trial. The objective of the trial was to assess the effect of tailored training of the heads of the households on the use of long lasting treated nets (ITN) on the burden of malaria in vulnerable groups. Detail description of the methods of the trial is given elsewhere [15]. In brief, 22 *Gots* (11 interventions and 11 controls) were selected and at least two ITN were distributed to each household in all *Gots* (villages). All of the heads of the households in the intervention villages were trained about the proper use of Insecticide-treated Nets (ITN). The proper use of ITN has been properly monitored in each household by trained village residents. The trained village residents also monitor the occurrence of malaria in each household in the intervention and control villages. To evaluate the effect of the intervention, mass blood investigation for the diagnosis of malaria and anaemia among all under-five children and pregnant women in the 22 study Gots has been undertaken three times a year. As part of the baseline survey, mass blood investigation and anthropometric measurements was done among 2410 under-five children in the study *Gots*.

Weight was measured using UNICEF electronic scale (Item No. 0141015 Scale mother/child, electronic) and height was measured using stadiometer (Holtain, UK). The nutritional status of children was determined using the International Reference Population defined by the U.S National Center for Health Statistics (NCHS) and Centres for Disease Control and Prevention [16]. Height-for-age (HAZ), weight-for-height (WHZ), and weight-for-age (WAZ) Z-scores were calculated based on this recommendation. Children were classified as stunting, wasting, and being under-weight if the HAZ, WHZ, and WAZ were <−2 standard deviation (SD). They were categorized as having severe stunting or wasting and being severe under-weight if the HAZ or WHZ, and WAZ were <−3 SD, respectively. Under-nutrition is defined as the presence of either stunting, wasting or under-weight.

For the diagnosis of malaria and anemia, a drop of blood from a finger prick was taken from the children. For malaria parasite identification, thick and thin films were prepared in the field and stained with Giemsa in Jimma Specialized Hospital. Each slide was read by experienced laboratory technicians. Absence of malaria parasite in 200 high power ocular fields of the thick film was considered as negative. Haemoglobin (Hb) concentration was determined using *HemoCue analyzer in the field (HemoCue Hb 301, Sweden)*. Anaemia and moderately severe anaemia were defined as Hb concentrations below <11.0 mg/dL and <7.0 mg/dL, respectively. Malaria was defined as any asexual parasitemia detected on a thick or thin blood smear.

Data were entered into computer, edited, cleaned, and analyzed using SPSS-12 software. To calculate the anthropometric indices, the data was exported to Epi Info 2000 software (version 2000, Atlanta, GA). Bivariate analysis was done to see the association between socio-demographic variables and malaria with under-nutrition. To control the effect of confounding variables, stepwise logistic regression was done.

The study has got ethical clearance from Jimma University and the WHO ethical committee. Written consent was obtained from caretakers of under-five children. Patients with anaemia, under-nutrition and malaria were given treatment by the health extension workers or the nearby health centres.

## Results

Nearly equal number of male (50.7%) and female (49.3%) children participated in the study. Infants (age less than one year) constituted 21% of the total children and one third of the children were above 47 months. The mean monthly family income of the children's family was 1232 Ethiopian Birr (ETHB) (SD±833). More than half of the family earned monthly income of above 1000 ETHB (**Table 1**).

**Table 1:** Socio-demographic characteristics of children in Gilgel Gibe Field Research Center, south-west Ethiopia (N = 2410).

Socio-demographic variables	Number	Percentage
**Age in months**		
<12	509	21.1
12–23	156	6.5
24–35	466	19.3
36–47	462	19.2
≥47	817	33.9
**Sex**		
Male	1223	50.7
Female	1187	49.3
**Birth order of children**		
1–3	1251	52.0
4–5	760	31.6
>5	394	16.4
**Site**		
Intervention	1207	50.1
control	1203	49.9
**Family monthly income (Ethiopian Birr)**		
<500	343	15.5
500–999	655	29.6
≥1000	1214	54.9

Significant proportion (40.4%) of the children were stunted (height-for-age<−2SD) and 18% of them were severely stunted. The prevalence of under-weight was 34.2%. One hundred and twenty two children (5.1%) were wasted (weight-for-height<−2SD). The prevalence of anemia was 32.4% and one tenth of the children had malaria parasite (**Table 2**).

**Table 2:** Nutritional status of children in Gilgel Gibe Field Research Center, south-west Ethiopia.

Nutritional status and malaria	Number	Percentage
**Weight-for-age(n = 2408)**		
Under-weight	824	34.2
Sever under-weight	232	9.6
**Height-for-age(n = 2408)**		
Stunted	973	40.4
Sever stunting	439	18.2
**Weight-for-height(n = 2408)**		
Wasted	122	5.1
Sever wasting	13	0.5
**Anaemia(n = 2410)**		
Haemoglobin, mean (±SD)	2410	11.50(1.66)
Haemoglobin<11 mg/dL (%))	780	32.4
Haemoglobin<7 mg/dL (%)	32	1.3
**Malaria parasite (n = 2410)**		
Yes	227	9.4
No	2183	90.6

There was no statistically significant association between malaria parasite and under-weight, [OR = 0.9, (95%CI: 0.7, 1.2)]. After controlling for the effect of potential confounding variables, children above one years of age were more likely to become under-weight as compared to infants. Sex, birth order and family income did not have statistically significant association with under-weight (**Table 3**).

**Table 3:** Factors associated with under-weight among children in Gilgel Gibe Field Research Center, south-west Ethiopia (N = 2410).

Variables	Weight-for-Age (WAZ)		Adjusted OR* (95% CI)	P-Value
	Not under-weight Number (%)	Under-weight Number (%)		
**Age in months**				
<12	394(77.4)	115 (22.6)	1	
12–23	104 (66.7)	52 (33.3)	1.7(1.1–2.5)	0.014
24–35	272(58.4)	194 (41.6)	2.4(1.8–3.2)	0.001
36–47	293 (63.2)	170 (36.8)	2.0(1.5–2.7)	0.001
≥47	524(64.1)	293 (35.9)	1.9(1.4–2.4)	0.001
**Sex**				
Male	790 (64.6)	433 (35.4)	1	
Female	796 (67.1)	391 (32.9)	0.9(0.7–1.1)	0.17
**Birth order**				
1–3	829 (66.3)	422 (33.7)	1	
4–5	496 (65.3)	264 (34.7)	1.0(0.9–1.3)	0.77
>5	257 (65.2)	137 (34.8)	1.1(0.9–1.4)	0.61
**Monthly Family income(Birr)**				
<500	231 (65.6)	112 (32.7)	1.0(0.8–1.3)	0.88
500–999	417 (63.3)	238(36.3)	1.2(1.0–1.5)	0.08
≥1000	819 (67.5)	395 (26.8)	1	
**Malaria parasite**				
Yes	153 (67.4)	74 (32.6)	0.9(0.7–1.2)	0.90
No	1433 (65.6)	750(34.4)	1	

*OR = Odds Ratio.

As the age of children increased, the prevalence of stunting increased. Compared to boys, girls were less likely to be stunted (OR = 0.8, 95% CI = 0.7–0.9). The other independent variables such as presence of malaria parasite, family income and birth order were not correlated with stunting (**Table 4**).

**Table 4:** Factors associated with stunting among children in Gilgel Gibe Field Research Center, south-west Ethiopia (N = 2410).

Variables	Height-for-Age (HA)		Adjusted OR* (95% CI)	P-Value
	Not stunted Number (%)	Stunted Number (%)		
**Age in months**				
<12	342(67.2)	167(32.8)	1	
12–23	79(50.6)	77(49.4)	2.1(1.4–3.1)	0.001
24–35	274(58.6)	192(41.2)	1.4(1.1–1.9)	0.01
36–47	267(57.9)	194(42.1)	1.5(1.1–2.0)	0.005
≥47	473(58.0)	343(72.0)	1.5(1.2–1.9)	0.001
**Sex**				
Male	700(57.2)	523(42.8)	1	
Female	735(62.0)	450(38.0)	0.8(0.7–0.9)	0.01
**Birth order**				
1–3	742(59.4)	508(40.6)	1	
4–5	455(59.9)	304(40.1)	1.0(0.9–1.3)	0.76
>5	234(59.4)	160(40.6)	1.0(0.8–1.3)	0.77
**Family income(Birr)**				
<500	201(58.8)	141(41.6)	1.1(0.9–1.4)	0.45
500–999	392(59.8)	263(40.2)	1.0(0.9–1.3)	0.61
≥1000	737(60.8)	476(39.2)	1	
**Malaria parasite**				
Yes	141(62.1)	86(39.9)	0.9(0.7–1.2)	0.85
No	1294(59.3)	887(40.7)	1	

*OR = Odds Ratio.

Children in the age group of 12–23 months were 2.2 times more likely to develop wasting than infants,[OR = 2.2, (95%CI: 1,1, 4.4)]. There was no statistically significant association between malaria, sex, income and birth order of the children with wasting (**Table 5**).

**Table 5:** Factors associated with wasting among children in Gilgel Gibe Field Research Center, south-west Ethiopia (N = 2410).

Variable	Weight-for-Height (WH)		Adjusted OR* (95% CI)	P-Value
	Not wasted Number (%)	Wasted Number (%)		
**Age in months**				
<12	485(95.3)	24(4.7)	1	
12–23	140(89.7)	16(10.3)	2.2(1.1–4.4)	0.03
24–35	445(95.5)	21(4.5)	0.9(0.5–1.7)	0.77
36–47	440(95.4)	21(4.6)	0.9(0.5–1.8)	0.97
≥47	776(95.1)	40(4.9)	1.1.(0.6–1.8)	0.84
**Sex**				
Male	1157(94.6)	66(5.4)	1	
Female	1129(95.3)	56(4.7)	0.9(0.6–1.3)	0.47
**Birth order**				
1–3	1181(94.5)	69(5.5)	1	
4–5	724(95.4)	35(4.6)	0.8(0.5–1.3)	0.43
>5	376(95.4)	18(4.6)	0.7(0.4–1.2)	0.19
**Family income(Birr)**				
<500	329(96.2)	13(3.8)	0.7(04–1.2)	0.19
500–999	622(95.0)	33(5.0)	0.9(0.6–1.4)	0.70
≥1000	1148(94.6)	65(5.4)	1	
**Malaria parasite**				
Yes	220(96.9)	7(3.1)	0.6(0.2–1.3)	0.18
No	2066(94.7)	115(5.3)	1	

*OR = Odds Ratio.

Children who had malaria parasite were 1.5 times more likely to become anaemic as compare to children who had no malaria parasite, [OR = 1.5, (95% CI: 1.1–2.0)]. Children above 12 months of age were less likely to become anaemic as compared to infants (**Table 6**).

**Table 6:** Factors associated with anemia among children in Gilgel Gibe Field Research Center, south-west Ethiopia (N = 2410).

Variables	Presence of anemia		Adjusted OR* (95% CI)	P-Value
	No, no (%)	Yes, no (%)		
**Age in months**				
<12	230 (45.2)	279 (54.8)	1	
12–23	102 (65.4)	54 (34.6)	0.4(0.3–0.6)	0.001
24–35	310 (66.5)	156 (33.5)	0.4(0.3–0.6)	0.001
36–47	318 (68.8)	144 (31.2)	0.4(0.3–0.5)	0.001
≥47	670 (82.0)	147 (18.0)	0.2(0.1–0.2)	0.001
**Sex**				
Male	830 (67.9)	393 (32.1)	1	
Female	800 (67.4)	387 (32.6)	1.0(0.8–1.2)	0.94
**Birth order**				
1–3	845(67.5)	406 (32.5)	1	
4–5	527 (69.3)	233 (30.7)	0.9(0.7–1.1)	0.31
>5	253 (64.2)	141 (35.8)	1.1(0.9–1.5)	0.56
**Family income(Birr)**				
<500	240 (70.0)	103 (30.0)	0.8(0.6–1.1)	0.20
500–999	452 (69.0)	203 (31.0)	0.9(0.7–1.1)	0.16
≥1000	799 (65.8)	415 (34.2)	1	
**Malaria parasite**				
Yes	140 (61.7)	87 (38.3)	1.5(1.1–2.0)	0.014
No	1490(68.3)	693 (31.7)	1	

*OR = Odds Ratio.

## Discussion

We have assessed the magnitude of under-nutrition, anemia and malaria and the interaction of malaria with under-nutrition and anaemia using a large sample size in under-five children at risk of malaria. The magnitude of stunting and under-weight in this study is almost similar to the findings of the 2005 Ethiopian Demographic Health Survey which revealed that 47% of under-five children were stunted and 38% were under-weight [4]. The high prevalence of under-nutrition in our study can be explained by the high level of food insecurity in the area [17] and lack of knowledge of the care givers to provide balanced diet to their children. Our finding of stunting and under-weight is also consistent with the findings of Hein et al in Vietnam who reported that 44% and 32% of under-five children were stunted and under-weight [18]. Phengxay and colleagues reported higher proportion of children(54%) had stunting and 35% under-weight in Laos [19].

In previous literatures, higher family size [20], maternal education [20], male gender [21], and poor feeding practices [22] were associated with under-nutrition of children. In our study, older children were more likely to have under-nutrition as compared to the younger ones which is similar to a previous report in Ethiopia [4]. As a result of short birth interval in the locality, care givers may give more attention to the younger children and neglect the older ones which predispose the later to malnutrition. Similar to other studies [21], [23], male gender was associated with stunting. Poor feeding practices may also contribute for under-nutrition of the children [4].

One third of the under-five children in this study had anemia which is comparable to the finding of Wolde et al in North Ethiopia [24]. One in ten of the children had malaria parasitemia. Detail description of the finding related to malaria is given elsewhere [15]. In this study, we did not find association between malaria and under-nutrition which is consistent with several previous studies [10], [11], [25]. Protein energy malnutrition might predispose children to malaria infection through reduction of malaria specific antibodies (IgG) [26]. Several previous reports indicated that deficiencies of micronutrients such as Vitamin A and Zinc are more important risk factors for the occurrence of malaria [5], [27]. In our study, deficiencies of these micronutrients might contribute for the occurrence of malaria. Malaria was strongly associated with anemia which is consistent with previous reports [5], [27], [28]. The interaction of malaria and anemia is complex. Malaria could cause anemia through cytokine mediated suppression of haematopoiesis or by predisposing the victim to other infection [5], [28]. Iron deficiency and parasite infestation can also contribute for the occurrence of anemia in our study. Previous report indicated that anemia in Ethiopia is primarily due to parasite infestation and malaria [24].

Although the study is the first of its kind in Ethiopia to assess the interaction of malaria and malnutrition in under-five children at risk of malaria, it has several limitations. First, we didn't assess level of micronutrients which might have impact on malaria morbidity. Second, several behavioural factors of malnutrition were not assessed. Third, cause effect relationship of under-nutrition and malaria could not be established.

In conclusion, under-nutrition and malaria are very common in under-five children around Gilgel Gibe Hydroelectric dam. Malaria was not associated with under-nutrition but strongly correlated with anemia. The Ministry of Health in collaboration with other partners should design nutritional and malaria intervention strategies for under-five children at risk of malaria. Children with malaria should be screened and treated for anemia.
